# AA amyloidosis associated with Castleman disease: A case report and review of the literature

**DOI:** 10.1097/MD.0000000000018978

**Published:** 2020-02-07

**Authors:** Luca Bernabei, Adam Waxman, Gabriel Caponetti, David C. Fajgenbaum, Brendan M. Weiss

**Affiliations:** a Penn Amyloidosis Program, Abramson Cancer Center; b Department of Pathology and Laboratory Medicine; c Division of Translational Medicine and Human Genetics, Perelman School of Medicine, University of Pennsylvania, Philadelphia, PA.

**Keywords:** AA amyloidosis, Castleman disease, lymph node hyperplasia, unicentric Castleman

## Abstract

**Rationale:**

AA amyloidosis (AA) is caused by a wide variety of inflammatory states, but is infrequently associated with Castleman disease (CD). CD describes a heterogeneous group of hematologic disorders that share characteristic lymph node histopathology. CD can present with a solitary enlarged lymph node (unicentric CD, UCD) or with multicentric lymphadenopathy (MCD), constitutional symptoms, cytopenias, and multiple organ dysfunction due to an interleukin-6 driven cytokine storm.

**Patient Concerns:**

We are reporting a case of a 26-year-old woman with no significant past medical history who presented with a 3-month history of fatigue and an unintentional 20-pound weight loss.

**Diagnosis:**

A CT-scan of the abdomen and pelvis revealed hepatosplenomegaly and a mesenteric mass. Congo Red staining from a liver biopsy showed apple-green birefringence and serum markers were suggestive of an inflammatory process. Post-excision examination of the resected mass revealed a reactive lymph node with follicular hyperplasia with kappa and lambda stains showing polyclonal plasmacytosis consistent with CD.

**Interventions:**

The patient underwent surgery to remove the affected lymph node.

**Outcomes:**

IL-6, anemia, leukocytosis, and thrombocytosis resolved or normalized 2 weeks after resection; creatinine normalized 9 months postsurgery. Twenty two months post-surgery her IFN-γ normalized, her fatigue resolved, her proteinuria was reduced by >90% and she had returned to her baseline weight.

**Lessons:**

Our case and literature review suggest that patients presenting with UCD or MCD along with organ failure should prompt consideration of concurrent AA amyloidosis.

## 1 Introduction

Amyloidosis is a rare group of diseases characterized by the deposition of amyloid fibrils in various tissues.^[1,2]^ The type and frequency of organ involvement varies with the subtype of amyloidosis as defined by the precursor protein, with the most common sites including the heart, liver, kidneys, and peripheral nervous system. Serum amyloid A (SAA) protein is the precursor protein in AA amyloidosis (AA). Production of SAA is induced by inflammatory mediators such as TNF-alpha, IL-1, and IL-6 that are chronically and persistently elevated in a wide range of chronic inflammatory diseases, including Castleman disease (CD).^[3,4]^ Treatment of AA amyloidosis is directed at controlling or eliminating the underlying inflammatory disease.

Castleman disease represents a rare group of lymphoproliferative disorders, which demonstrate a spectrum of histopathological features ranging from hyaline vascular sub-type to plasmacytic sub-type.^[5]^ CD can present with either unicentric (UCD) lymphadenopathy and mild inflammatory symptoms or multicentric (MCD) lymphadenopathy with constitutional symptoms, cytopenias, and multiple organ dysfunction. Human herpes virus-8 (HHV-8) is the etiological driver of approximately 50% of MCD cases, whereas the etiology is unknown for the remaining HHV-8-negative/idiopathic MCD (iMCD) cases.^[6]^ The pathophysiology of UCD and iMCD is poorly understood. UCD is thought to arise via a neoplastic process most likely involving follicular dendritic cells.^[7]^ Multiple processes have been hypothesized to play a role in iMCD including neoplastic cells, autoinflammation/autoimmunity, and infection.^[7]^ Though the etiology of iMCD is unknown, interleukin-6 (IL-6) is the pathological driver in a large portion of cases. Prior reports have shown that excess IL-6 in UCD and iMCD^[8]^ can lead to increased transcription of SAA^[9]^ and the development of AA amyloidosis as a rare complication.

Treatment and outcomes differ greatly across CD. Surgical resection is highly effective for UCD with a 95% 10-year overall survival rate.^[10]^ B cell depletion with rituximab induces durable remission in nearly all HHV-8-associated MCD cases, leading to a 92% 5-year overall survival.^[11]^ The anti-IL-6 therapy, siltuximab, is effective in about one-third of iMCD patients and considered front-line treatment. There is no standard approach for iMCD patients who fail siltuximab, but therapeutic options include cytotoxic chemotherapy, immunosuppressants/immunomodulators, and corticosteroids. As such, iMCD patients have a relatively worse 5-year overall survival of 50% to 77%.^[12–15]^

Here, we present a case report and review of the literature of amyloidosis associated with CD.

## 2 Case report

**Figure 1:**
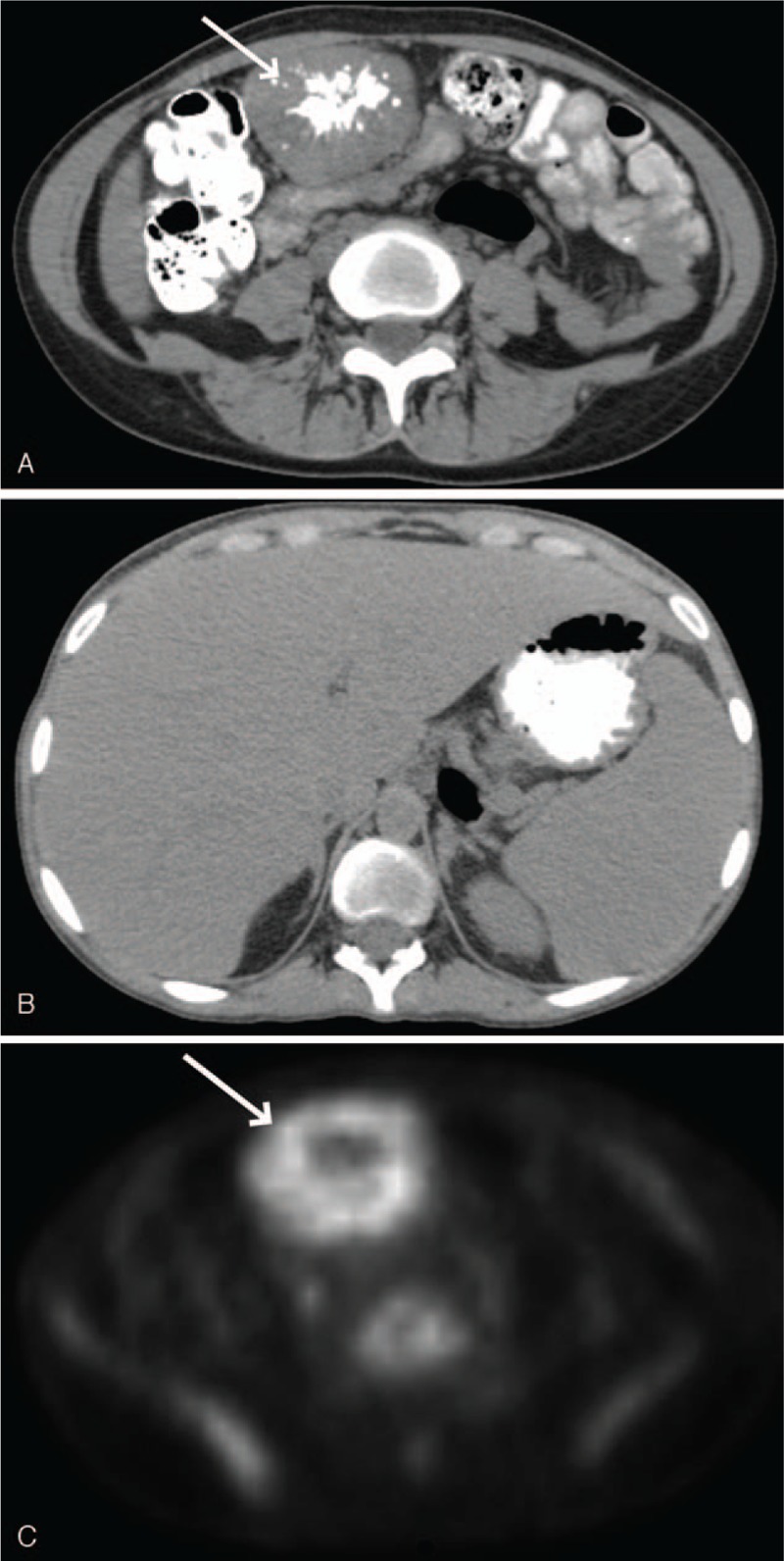
CT scan of the abdomen showing mesenteric mass (a; top), non-FDG avid hepatosplenomegaly (b; middle) and PET showing FDG avidity of the mass (c; bottom).

A 26-year-old woman with no significant past medical history presented with a 3-month history of fatigue and an unintentional 20-pound weight loss. Initial laboratory evaluation revealed a white blood count of 22,000 cells/μl (normal range: 4000–11,000 cells/μl), a hemoglobin of 7.3 g/dl (normal range: 12.0–16.0 g/dl), and a platelet count of 713,000/μl (normal range: 150,000–400,000/μl). Her prothrombin time was prolonged at 16.6 seconds (normal range: 12.2–14.2 seconds) and her activated partial thromboplastin time was prolonged at 40.4 seconds (normal range: 20.8–34.4 seconds). Flow cytometry performed on peripheral blood was negative for a lymphoid neoplasm, and BCR-ABL and JAK2 mutation analyses, also done on peripheral blood were negative as well. She subsequently developed acute abdominal pain and had a CT-scan of the abdomen and pelvis that revealed marked hepatosplenomegaly and a mesenteric mass (Fig. 1). She had an incomplete biopsy of the mesenteric mass that showed non-diagnostic necrotic material and a liver biopsy that demonstrated eosinophilic material in the sinusoids with apple-green birefringence on Congo-red staining. These biopsies were complicated by hemoperitoneum requiring packed red blood cell transfusion. A 24-hour urine collection revealed 2.8 g of protein consisting almost entirely of albumin. A bone marrow biopsy and aspirate revealed 10% polytypic plasma cells. Serum protein electrophoresis showed polyclonal hypergammaglobulinemia, quantitative immunoglobulins showed elevated levels of IgG and IgA, and serum free light chain measurement showed free kappa light chains of 178.5 mg/L and free lambda light chains of 156.5 mg/L (ratio 1.14). A coagulation workup revealed a mild reduction in Factor V to 60%. A cytokine panel revealed elevation of IL-6 to 54 pg/ml (normal range: 0–12 pg/ml) and IFN-γ of 14 pg/ml (normal range: 0–7 pg/ml). Mass spectrometry performed on the liver biopsy revealed AA-subtype amyloidosis.

**Figure 2:**
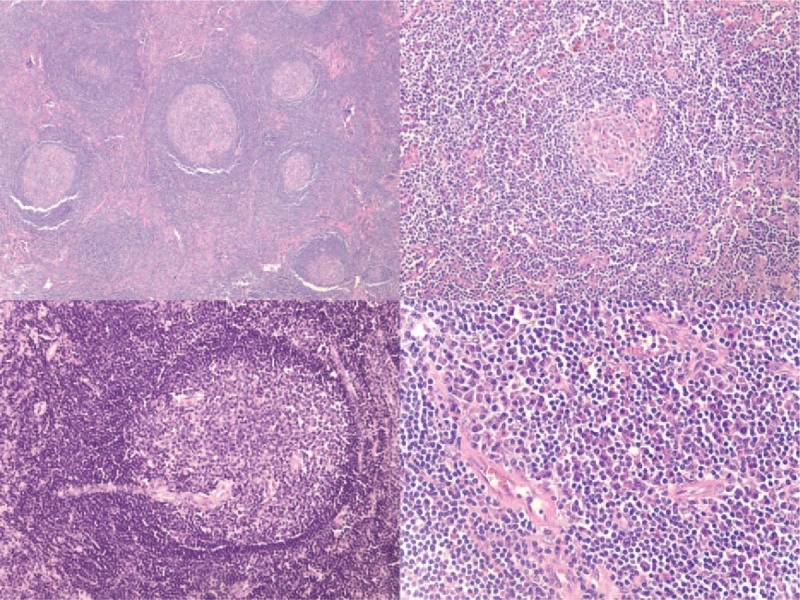
H&E-stained slides of the resected mass revealed features of reactive follicular hyperplasia (a; top left) with few regressed and sclerotic germinal centers surrounded by ill-defined mantle zones (b; top right), very rare germinal centers with radially-penetrating vessels (c; bottom left) and prominent interfollicular plasmacytosis with morphologically unremarkable plasma cells (d; bottom right).

**Figure 3:**
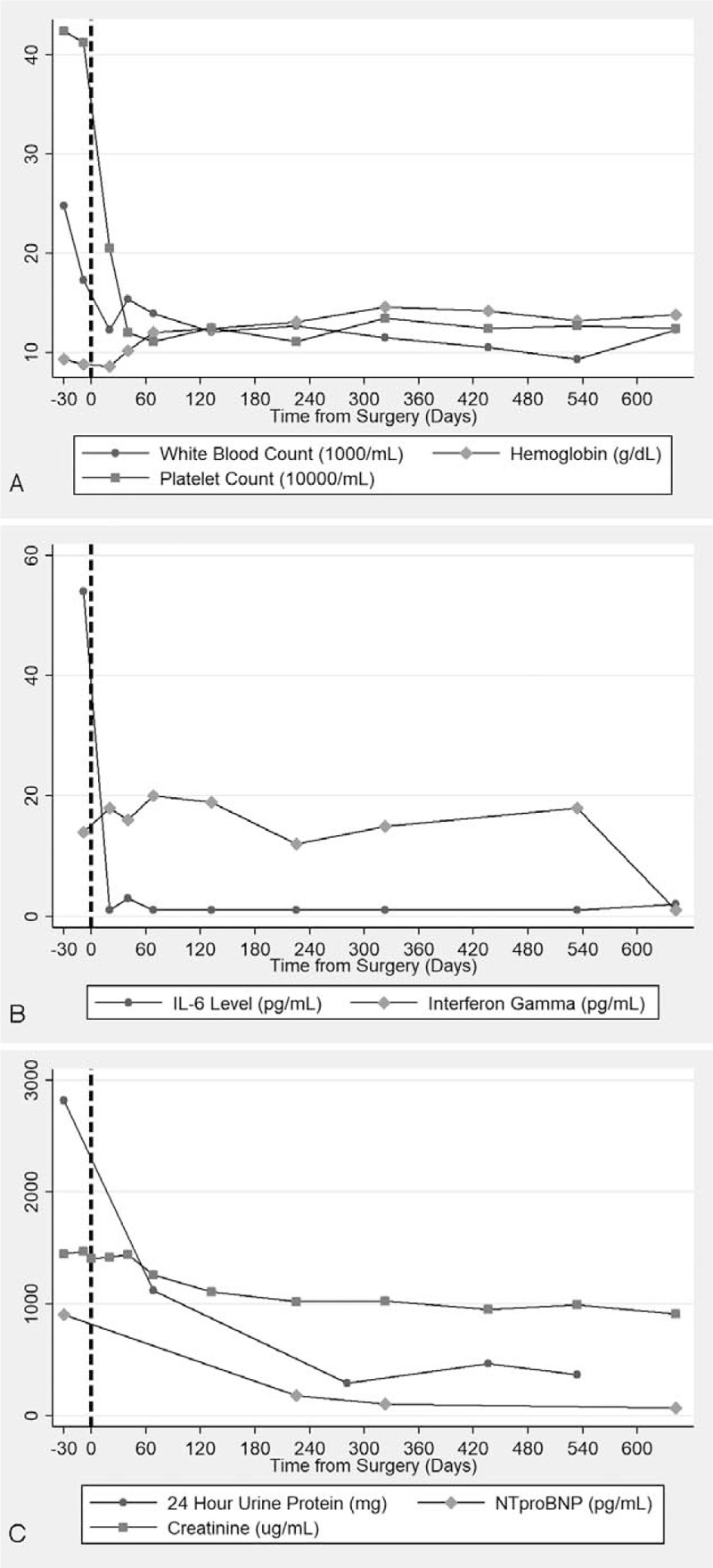
Baseline and response to surgery of blood counts (a; top), IL-6 and IFN-γ levels (b; middle) and 24-hour protein excretion and NT-proBNP levels (c; bottom).

A PET/CT revealed no additional sites of disease outside the abdomen (Fig. 1). She proceeded to a laparoscopic resection of the mass. Pathology (Fig. 2) revealed a reactive lymph node with follicular hyperplasia with kappa and lambda stains showing polyclonal plasmacytosis consistent with CD. A comprehensive rheumatologic, oncologic, and infectious workup ruled out alternate causes of these histopathologic findings. She was diagnosed with a rare case of HHV-8 negative UCD plasma cell variant, complicated by AA amyloidosis with renal and hepatic involvement. Within 2 weeks following complete lymph node excision, there was normalization of IL-6 level and resolution of her leukocytosis, anemia, and thrombocytosis; proteinuria and NT-proBNP reduced within 2 months of surgery (Fig. 3). At 9 months postsurgery her creatinine had normalized and at 22 months her IFN-γ had also normalized. Her fatigue resolved, proteinuria reduced by >90%, and she returned to her baseline body weight.

## 3 Discussion

Considering this rare association and the excellent outcome of our patient following appropriate treatment of the underlying UCD, we performed a review of all published cases of UCD (N = 39, Table 1) and MCD (N = 22, Table 2) complicated by AA amyloidosis. For UCD patients, the median reported follow-up time was 22 months and for MCD cases the median follow-up was 12.5.

**Table 1:**
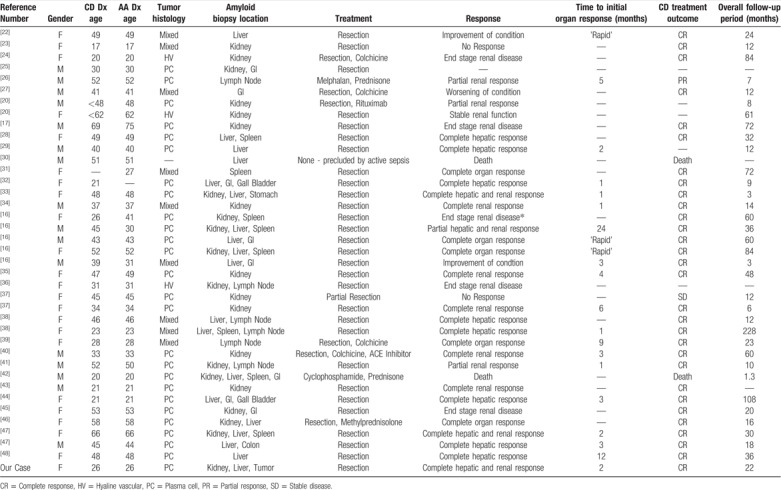
Cases of UCD associated with AA published in literature.

**Table 2:**
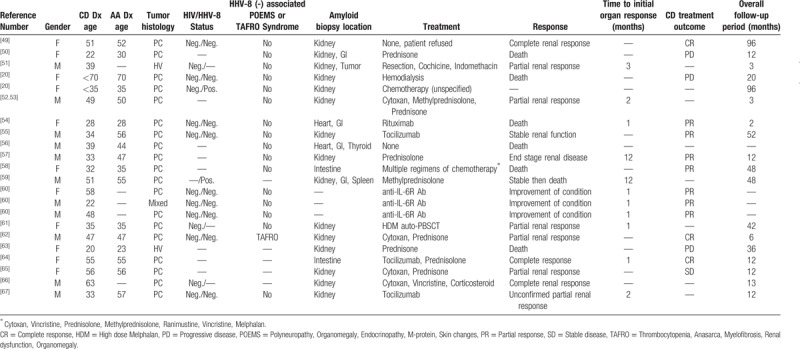
Cases of MCD associated with AA published in literature.

### 3.1 UCD associated with AA amyloidosis

The median age of these patients was 42% and 59% were female. The UCD histopathological sub-type was plasmacytic in 67%, mixed in 23%, and hyaline vascular in 8%. The diagnosis of AA amyloidosis was made by biopsy of the kidney in 20 cases (51%), liver in 12 cases (31%), lymph node in 2 cases (5%), gastrointestinal tract in 1 case (3%), spleen in 1 case (3%) and gallbladder in 1 case (3%). Amyloid was confirmed in more than 1 organ in 51% of cases. The diagnosis of AA amyloidosis complicating UCD was made concurrently with initial diagnosis of UCD in 72% of patients, prior to diagnosis of UCD in 10% and following diagnosis of UCD in 13%. The kidney was the organ most frequently involved, which was seen in 59% of cases. Additional organ involvement included the liver in 49%, gastrointestinal tract in 21%, lymph node in 15%, and spleen in 15% of patients. In 34 of 39 UCD cases, complete surgical resection of the UCD lymph node was achieved, leading to at least partial improvement in organ function in 27 of those cases. Complete organ response occurred in 54% of cases and 2 of 37 patients where the outcome was detailed died. Five patients’ clinical courses were complicated by end stage renal disease (ESRD) after resection, 4 of which occurred progressively and 1 case where ESRD resulted from postoperative sepsis.^[16]^ Initial improvement was often rapid and within 1 month of resection. In 2 cases, resection was considered but was not feasible due to the patient medical condition. Improvement of organ function appeared to be related to the extent of tissue injury prior to treatment of the underlying UCD. Interestingly, Eroglu et al described a patient diagnosed with AA amyloidosis 6 years after complete surgical resection of UCD.^[17]^ The authors attributed the presence of amyloidosis to systemic low-level inflammation despite complete resection of UCD, but an alternative etiology cannot be ruled out.

### 3.2 MCD associated with AA amyloidosis

Nine out of 22 MCD cases were reported to be HHV-8-negative/iMCD, 2 cases were HHV-8-positive, and HHV-8 status was not reported for 11 cases. The median age of all MCD cases was 47, and 50% were female. The MCD histological subtype was plasmacytic in 86%, hyaline vascular in 9% and mixed in 5%. The diagnosis of AA amyloidosis was made by biopsy of the kidney in 15 cases (68%), gastrointestinal tract in 3 cases (18%), and the heart in 1 case (9%). Amyloid in more than 1 organ was confirmed in 22% of cases. Of cases where this information was available, the diagnosis of AA amyloidosis complicating MCD was made concurrently with initial diagnosis of MCD in 23% of cases, following the diagnosis of MCD in 55% of cases and in no cases prior to diagnosis of MCD. Among patients with MCD, 11 of 22 patients had at least a partial improvement of organ function with treatment. Seven of 12 (58%) patients treated with combination chemotherapy showed symptomatic improvement and lymph node regression, whereas 6 of 7 (86%) patients treated with monoclonal antibodies directed at B cells or IL-6 showed similar responses. In total, 6 of 21 patients where the outcome was reported died. We expect the continued increase in use of these monoclonal antibody therapies, such as tocilizumab, siltuximab, and rituximab will greatly improve outcomes for MCD patients, particularly those with associated AA amyloidosis.

Our case and literature review suggest that patients presenting with UCD or MCD along with organ failure should prompt consideration of concurrent AA amyloidosis. Lee et al reported that amyloidosis was the most common renal disease in CD,^[18]^ Yuan et al reported amyloidosis as the most common renal histological finding in their series of CD cases,^[19]^ and Karoui et al reported that amyloidosis was the second most common cause of renal disease after small vessel lesions (SVL) in their CD case series;^[20]^ cases of CD with amyloidosis were more severe than CD cases with SVL. Given that kidney disease was the most common manifestation of AA amyloidosis associated with UCD and MCD, proteinuria and/or renal failure should raise the index of suspicion. The ability to achieve control of the underlying CD is associated with a greater chance of organ improvement as illustrated by the much higher rates of complete organ function improvements in UCD, which can be cured with lymph node excision, compared to MCD.

A recent proteomic analysis of 1129 analytes in paired flare-remission plasma samples from 6 iMCD patients revealed that SAA was 1 of the most up-regulated proteins between flare and remission.^[21]^ None of these patients had documented AA amyloidosis, but most demonstrated hepatic and renal dysfunction of undiagnosed etiology. It is possible that these cases and others with iMCD may have underlying AA amyloidosis.

It is likely that recent advances in the treatment of HHV-8-associated MCD and iMCD will have a major impact in AA amyloidosis complicating MCD.

## Author contributions

**Data curation:** Luca L. Bernabei.

**Formal analysis:** Luca L. Bernabei.

**Investigation:** Adam J. Waxman, Gabriel Caponetti, Brendan M. Weiss.

**Writing – original draft:** Luca L. Bernabei, Adam J. Waxman.

**Writing – review & editing:** Gabriel Caponetti, David C. Fajgenbaum, Brendan M. Weiss.

Luca L. Bernabei orcid: 0000-0002-0213-8914.
